# A national evaluation of QbTest to support ADHD assessment: a real-world, mixed methods approach

**DOI:** 10.1186/s12913-024-11693-7

**Published:** 2024-10-08

**Authors:** Sophie S. Hall, Caitlin McKenzie, Louise Thomson, Benji-Rose Ingall, Madeleine J. Groom, Nicole McGlennon, Mark Dines-Allen, Charlotte L. Hall

**Affiliations:** 1 Nottingham Clinical Trials Unit, School of Medicine, University of Nottingham, Nottingham, UK; 2 Nottinghamshire Healthcare NHS Foundation Trust, Nottingham, UK; 3 School of Medicine, Academic Unit of Mental Health & Clinical Neurosciences, University of Nottingham, Nottingham, UK; 4 NIHR Nottingham Biomedical Research Centre, Nottingham, UK; 5 NIHR MindTech HealthTech Research Centre, School of Medicine, University of Nottingham, Nottingham, UK; 6 Health Innovation East Midlands, University of Nottingham, Nottingham, UK

**Keywords:** ADHD, QbTest, Real-world evaluation, Case-note, Qualitative, Assessment

## Abstract

**Background:**

QbTest is a commercially available, computerised test of attention, impulsivity, and activity designed to assist with the diagnosis of attention deficit hyperactivity disorder (ADHD). Health Innovation East Midlands (formerly East Midlands AHSN), led the implementation of the QbTest on behalf of the 15 Health Innovation Networks across Child and Adolescent Mental Health services (CAMHS) and Paediatric sites in England between April 2020 and March 2023. We evaluate the impact of this programme on diagnostic assessment at participating sites.

**Methods:**

A mixed-methods approach was used including: case-note data collected on 10–30 cases per site pre and post QbTest implementation; interviews with healthcare staff working with QbTest; and surveys to explore perspectives of healthcare staff and patients/carers. Case-note data was descriptively analysed to compare time to diagnosis (number of appointments and days) pre/post QbTest implementation. Survey data was analysed descriptively. Qualitative interview data was explored using thematic analysis.

**Results:**

Case-note data was provided by 20 sites across England. Comparison of mean values pre- and post-QbTest implementation identified a decrease of 0.37 (11.5%) in number of appointments to reach a diagnostic decision, a 55-day (12.5%) increase in days from initial referral to diagnosis, and a 12-day (10.3%) increase in days to reach a diagnostic decision. Exploratory analyses indicated greater benefit for Paediatric services over CAMHS, in terms of a decrease in days from referral to diagnosis and number of appointments to diagnosis.

Interviews with healthcare staff (*n*=21) revealed that the QbTest was perceived to support a faster, more efficient diagnostic process. Survey data (*n*=65 healthcare staff, *n*=22 patients/carers) identified that the QbTest helped patients understand their symptoms and the diagnostic decision.  Although some logistical issues (e.g., room requirements) and patient issues (e.g., sensory sensitivity) were identified, healthcare staff considered that QbTest was easily incorporated into the ADHD assessment pathway.

**Conclusion:**

The national implementation of QbTest in ADHD clinics resulted in a small reduction in the number of clinical appointments needed to reach a diagnostic decision, with greatest benefit demonstrated in Paediatric sites. Data were impacted by COVID-19 therefore, further evaluation is warranted.

**Supplementary Information:**

The online version contains supplementary material available at 10.1186/s12913-024-11693-7.

## Background

Attention-deficit/hyperactivity disorder (ADHD) is a common neurodevelopmental disorder characterised by three core symptom domains: inattention, hyperactivity, and impulsivity. An estimated 4-8% of children in the UK have ADHD [1], however, the exact figures could be higher due to underdiagnosing. Although once considered a childhood condition, there is increasing recognition that ADHD continues into adulthood [2].

The symptoms of ADHD can impact several areas, including school, family life, and personal life [3–5]. Children with probable ADHD are more likely to be below expected attainment in literacy and numeracy, have poorer school attendance, and be identified as having special educational needs (SEN) [6]. Furthermore, untreated ADHD that continues into adulthood is associated with an increased risk of car accidents, higher rates of divorce and substance misuse, more frequent job changes, and suicidality [7, 8]. Given the impact of ADHD, it is crucial that children with probable ADHD have timely access to diagnosis and treatment.

Given the variation in causes and behavioural consequences of ADHD, there is no single test used to diagnose the disorder, and the clinician’s judgment is the most widely accepted method of assessment. For the clinician to make an informed decision on the diagnosis of ADHD, they will generally gather information from the caregiver, teachers, the child themselves (where appropriate), make clinical observations, conduct school observations, and conduct standardised tests [1]. However, this approach is reliant on subjective methods; these can be lengthy to conduct, difficult to interpret, and frequently contradictory [9]. More recently, there has been increasing interest in the use of more objective computerised testing to streamline and support this process [10–12] In the UK and Europe, perhaps the most widely used neurocognitive computerised assessment of ADHD is QbTest (Qbtech Ltd).

QbTest is a commercially available product that combines a computerised continuous performance test (CPT) to measure attention and impulsivity with a high-resolution infrared motion-tracking system to measure motor activity. Test results for each domain are visually represented in a report, which also provides summary scores for the individual based upon their deviation from average scores for their age and sex group [12, 13]. QbTest is a CE Marked Class 1 medical device, and the US Food and Drug Administration (FDA) has cleared QbTest as a decision-aid tool to augment, but not replace, standard clinical assessment of ADHD. QbTest is not a standalone diagnostic tool, and a recent meta-analysis confirms the test should not be used as such [14]. However, when used appropriately, alongside other ADHD assessment tools, QbTest may produce service efficiencies to facilitate the assessment process [14].

The clinical benefits of QbTest were evaluated in a randomised controlled trial ‘AQUA-Trial’ [13]. Clinicians who had access to a QbTest report to supplement their clinical decision making were 40% faster in reaching a diagnostic decision about the presence or absence of ADHD than those who did not have access to a QbTest report. Further analysis revealed clinicians were more likely to make a diagnostic decision within 6 months (76% of patients received a diagnostic decision vs. 50% of patients for whom clinicians did not receive a QbTest report) [15]. Furthermore, appointment length when utilising the QbTest was reduced by 15%, demonstrating clear potential for efficiency savings. Qualitative evaluations of QbTest demonstrate that the test is acceptable and feasible to both clinicians and families [16].

Following the AQUA trial, the Health Innovation East Midlands (formerly known as East Midlands Academic Health Science Network) supported the implementation of QbTest across seven NHS sites, in three East Midlands NHS Trusts. The AHSN is a UK government funded initiative to support the spread of innovation into routine healthcare [17]. In 2018 the HIEM reported audit data (a systematic process used to evaluate patient care) collected pre and post QbTest implementation in seven NHS sites [18]. Their data revealed that QbTest reduced time to diagnosis by an average of 153 days. Their data also revealed that QbTest reduced the number of appointments required to make a diagnosis, saving up to one-third of clinical time through less appointments. Cost analysis revealed that QbTest resulted in a return on investment (ROI) to the NHS of £84,460 (median result), equating to an ROI of £5.97 for every £1 spent. Costs included when calculating the ROI when using QbTest included the cost per use, staff time to carry out the test, and equipment costs. QbTest includes training as part of their overall package to their customers, with no attributable costs. Although these findings were not published, a summary of the evaluation can be found here https://healthinnovation-em.org.uk/our-work/innovations/focus-adhd, including contact details for further information.

As a result of the clinical and cost effectiveness of QbTest demonstrated in the AQUA trial and the regional service evaluation, QbTest was rolled out nationally in 2020 with support from the 15 Health Innovation Networks across the UK. To facilitate efficient service implementation and to establish whether similar impacts would be observed when evaluated at scale, a national service evaluation was warranted.

### Aim

This study aimed to provide an independent evaluation of the impact of a national roll-out of QbTest supported by the Health Innovation Networks. Specifically, the study examined the impact of implementing QbTest on number of appointments and time required to reach a confirmed ADHD diagnostic decision (diagnosis ruled in or out from initial assessment to confirmed diagnosis), and the acceptability and feasibility of QbTest implementation as perceived by patients/caregivers and clinicians (qualitative exploration). The length of time and number of appointments to reach diagnosis reflect potentially important implications for improving healthcare efficiency through reducing clinician time per case load and may reduce waiting list time by speeding up participant flow. Assessing patient and clinician acceptability and feasibility is essential for delivering patient-centred care, ensuring clinical effectiveness, promoting adherence and driving quality improvement in healthcare delivery. Qbtech Ltd were not involved in the design or conduct of the evaluation, supporting an unbiased evaluation of the rollout.

#### Methods

Ethical approval was granted by University of Nottingham, Faculty of Medicine and Health Sciences ethics committee (Ref; FMHS 299-0621) for the interview data. The evaluation took place in 2021-2022.

### Site selection

CAMHS and Paediatric services in England were invited to participate in the collection of case-notes, interviews, and survey data. Eligible services were those who (1) had QbTest implemented at their site after April 2019; (2) had undertaken ADHD assessments prior to and post QbTest implementation; and (3) were able to provide at least 10 cases pre- and post-implementation of QbTest. Sites who implemented QbTest pre-April 2019 were not included in the study because they were not part of the Health Innovation Network supported roll-out and therefore may have followed a different pathway to implementation to that which is currently used Our Study Advisory Group suggested including sites who could provide data on a minimum of 10 cases to help ensure QbTest was well-integrated as part of clinical practice.

### Case-note data

Case-note data was collected to explore changes in service efficiencies post QbTest implementation. Sites were asked to return a target of 30 cases with pre- and post- implementation data available, with an absolute minimum of 10. There was no difference between CAMHS and Paediatric sites on the number of cases provided.

Sites completed online training to support the collection of case-note data. The training was devised in collaboration with sites who were not eligible to participate in the study because they had implemented QbTest pre-April 2019. Sites were provided with a case-note template excel tool to support data collection. The data collected included: date of initial ADHD referral, date of first appointment with ADHD clinician, date of diagnosis, number of clinic appointments to diagnosis, and whether a school observation was conducted. These variables were all represented as column headings in the excel tool.

Sites were initially requested to return case-note data on five pre and five post implementation cases so any issues could be identified, and further training provided as necessary. This further training was provided via videoconferencing or telephone to discuss errors. Once all the available data was returned, full data sets were reviewed for completeness and accuracy and all data queries were resolved with sites prior to evaluation. Each site received an individual report on their data.

#### Analysis

Descriptive analysis including means, medians, and ranges were calculated for the pre- and post-implementation data to measure change.

The primary outcomes of interest were number of appointments and time required to reach a confirmed ADHD diagnostic decision (diagnosis ruled in or out). Time was measured in two ways: number of days to diagnosis from the first appointment and number of days to diagnosis from the date of the initial ADHD assessment referral. Number of appointments was calculated as the total number of in-person and telephone/video-conferencing consultations.

To understand the generalisability of the results, two unplanned sub-group evaluations were conducted. The first compared CAMHS sites with Paediatric sites due to differences in their service models that may have impacted implementation. The second compared sites with low (< 20 tests/month) vs. high rates (≥ 20 tests/month) of QbTest assessments, with the rationale that sites who used QbTest more regularly may be more likely to have QbTest better embedded in their service.

### Survey data

Survey data was collected to further explore acceptability and feasibility of implementation and the patient/caregiver experience. The survey was developed by the AQUA-Trial research team to gather opinions and perspectives of patients and clinicians on the QbTest [16]. Item generation was created by the multi-disciplinary AQUA study team, which includes patients and public members, clinicians and academics.

#### Clinician survey

Clinicians who attended the case-note training were provided with a link to the online survey and encouraged to share this widely with healthcare professionals within their team who used QbTest. The survey was hosted on Jisc (https://www.jisc.ac.uk/) and consisted of 13 questions pertaining to when in the ADHD pathway they administer the QbTest, and their perceptions of the value of the test, interpreting the test, and communicating with families. Responses were recorded using a five-point Likert scale from strongly agree to strongly disagree, with a mixture of positive and negatively worded questions. There was an additional open-ended question asking clinicians to reflect on which cases they find QbTest most helpful for. The survey took approximately five minutes to complete.

#### Patient/carer survey

Clinicians at participating sites invited caregivers of patients to complete an online survey at the end of the assessment process, regardless of whether an ADHD diagnosis had been confirmed as present or absent. The survey questions were aimed at the child completing the test. The survey could be completed by the caregiver with input from the child, or by the child alone. The survey consisted of five Likert-style questions (strongly agree to strongly disagree, with a mixture of positively and negatively worded items) and two open-ended responses pertaining to their perceptions of the utility of QbTest in understanding their symptoms, the diagnostic decisions which were made, and their experience. Participants read an information sheet and gave online consent to participate.

#### Analysis

Descriptive analyses were carried out using SPSS version 19. Open ended items were analysed using inductive thematic analysis.

### Interview data

Interview data was collected to explore acceptability and feasibility of implementing QbTest. Site staff from eligible sites were invited to take part in a semi-structured interview through an email invitation (see Appendix A for interview schedule). Interviews were conducted online. Staff were purposively recruited to represent a range of roles, including healthcare professionals who were responsible for delivering QbTest, clinicians who interpret the test, and managers who were responsible for the implementation of the test at their site. Staff were recruited until data saturation was reached (i.e., no new themes emerged)[19]. Interviews ranged from 30 to 60 min in length and staff were given a £20 online shopping voucher for participating.

#### Analysis

Interviews were transcribed and anonymised before being reviewed and coded. After familiarisation with the data, open coding was conducted on a proportion of the transcripts by two researchers. Codes were created by identifying passages of texts that were relevant to the research questions. Following discussion between two researchers, the initial list of codes were combined into a smaller set of codes. This set of codes was then applied to all transcripts, whilst new codes were also generated to reflect data that didn’t fit into the existing list of codes. Two researchers completed the coding of all the transcripts in this way. A third researcher independently coded 10% of the transcripts. One researcher developed the initial grouping of the codes into themes and sub-themes and these were discussed and finalised with the wider team. This process of finalising the themes and sub-themes was guided by the domains of the NASSS framework, which provided a super-ordinate structure which the themes were fitted into. Excel was used to support coding or analysis. The analysis sought to address the three research questions: (i) What is the patient experience of the QbTest? (ii) What is the clinician and service experience of using the QbTest?, (iii) Which factors are important as barriers and facilitators in the implementation of the QbTest into clinical settings? A Framework approach was used around these questions [20] in which a thematic analytic was adopted. Domains within the NASSS (non-adoption, abandonment, scale-up, spread, sustainability) framework [21] were used within the analysis to guide the development of themes relevant to the implementation of technological innovations in health care. The NASSS framework provides a holistic perspective on the challenges and complexities involved in the adoption and implementation of health technologies, helping researchers and practitioners identify potential barriers and facilitators at each stage of the process. As such, this framework was well-suited to address the study aim.

## Results

### Case-note data

**Fig. 1:**
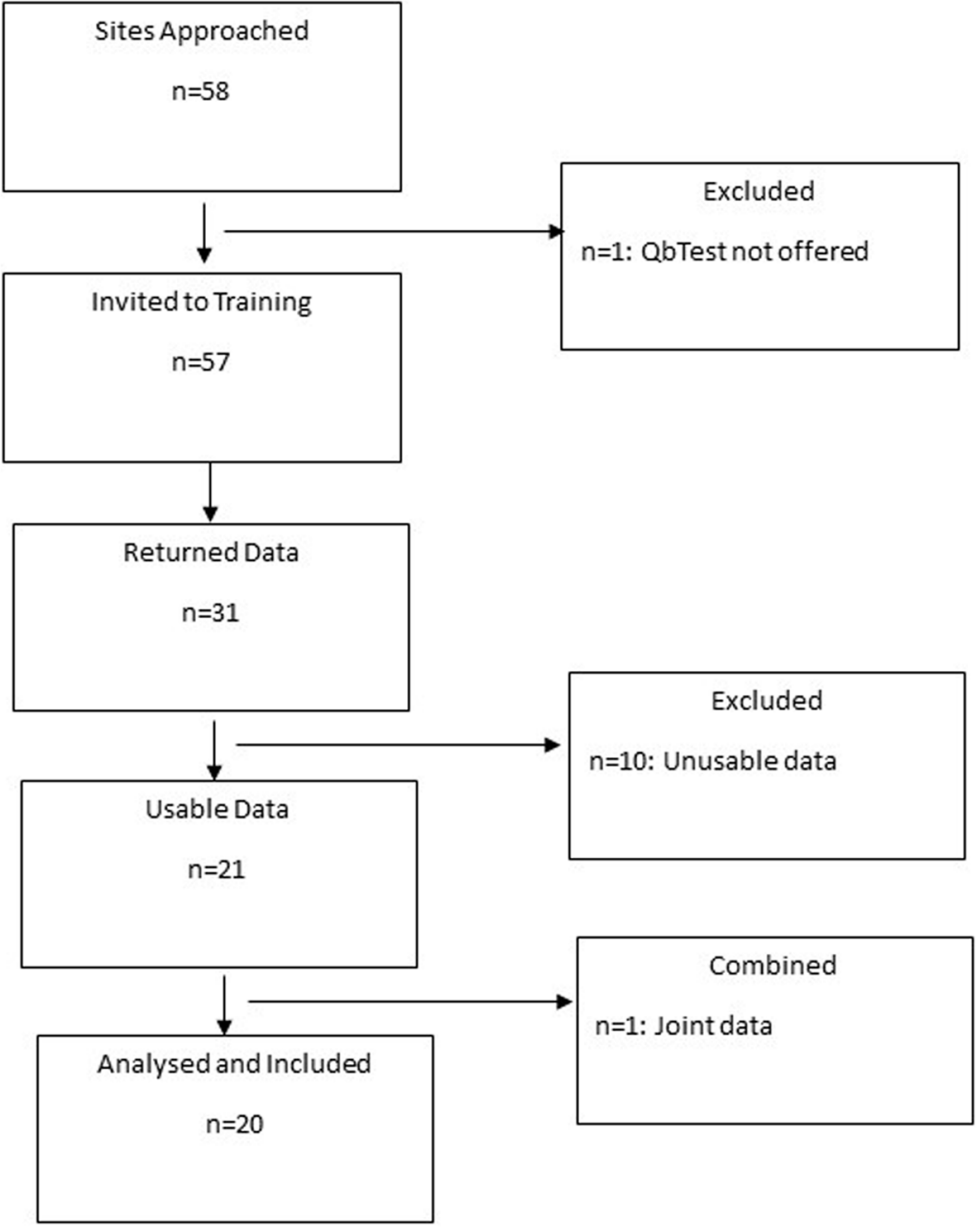
Flowchart of site recruitment

A total of 58 sites were invited to participate, 31 sites returned case-note data with 21 of these sites returning usable case-note data (two sites merged their data, which left 20 full datasets). Figure 1 illustrates the recruitment process. The most common reasons for sites deciding not to participate were capacity issues such as limited time/resources and staffing issues.

### Site characteristics

Most (*n* = 12) sites that returned usable case-note data were CAMHS, and seven were Paediatric services. One site operated both as a CAMHS and Paediatrics service and thus was not included in the comparison of CAMHS and Paediatric services. Geographically, most participating sites were located in the South-East and London (six and five sites, respectively), three sites were from the Yorkshire/Humber region, two sites from the East Midlands, two sites from the South-West, one site from the West Midlands, one from the North-East and one from the Anglia region.

A total of 13 sites provided a full dataset of 30 pre and post cases. The lowest number of data returned from a site was 10 pre and 10 post implementation cases. In total, there were 549 pre-implementation cases, and 549 post-implementation cases analysed. One site used the QbTest for cases which initially presented as complex(e.g., multiple co-occurring conditions were mentioned in the referral letter), and the remainder used the test for all patients. The QbTest was utilised early in the ADHD assessment pathway at all sites, however, a small number of cases (36 cases) from multiple sites utilised the QbTest later in the pathway. Most patients (93%) had the QbTest carried out as a QbTest only appointment, with no other assessments conducted during the appointment. In most cases (93%), the QbTest results were available to discuss with a clinician at the patients next clinical appointment. There was a 5% increase in patients having ADHD ruled out as a diagnosis post QbTest implementation compared to pre implementation.

#### Time and appointments to diagnosis

Table 1 reports descriptive statistics showing pre-QbTest and post-QbTest implementation data, with an 11.5% reduction in the number of clinical appointments pre to post, and a 12.2% increase in the number of days from referral to diagnosis.

**Table 1:** Mean (range) descriptive statistics for the primary outcome measures across different types of sites

Site Type	Pre-QbTest Implementation			Post-QbTest Implementation			Percent Change		
	*No of appointments*	*Days: referral to diagnosis*	*Days: 1st appointment to diagnosis*	*No of appointments*	*Days: referral to diagnosis*	*Days: 1st appointment to diagnosis*	*No of appointments*	*Days: referral to diagnosis*	*Days: 1st appointment to diagnosis*
All sites	3.22 (1–50)	452 (15-3276)	117 (0-1570)	2.85 (1–32)	507 (43-1821)	129 (0-1378)	-11.5%	+ 12.2%	+ 10.3%
CAMHS (*n* = 326)^a^	4.13 (1–50)	442 (18-1161)	119 (0-888)	3.75 (1–32)	566 (43-1821)	135 (0-1378)	-9.2%	+ 28.1%	+ 13.4%
Paediatrics (*n* = 194)	2.01 (1–15)	444 (15-3276)	130 (0-1570)	1.63 (1–4)	367 (62-1494)	138 (0-1036)	-18.9%	-17.3%	+ 6.2%
Frequent testers (*n* = 146)	5.27 (1–50)	422 (15-1090)	101 (0-582)	3.86 (1–32)	391 (62-1125)	152 (0-656)	-26.8%	-7.3%	+ 50.5%
Less frequent testers (*n* = 403)	2.48 (1–15)	464 (18-3276)	123 (0-1570)	2.49 (1–17)	552 (43-1821)	121 (0-1378)	+ 0.4%	+ 19.0%	-1.6%

^a^*n* = number of cases included in the case-note data

#### Comparing CAMHS with Paediatric services

Prior to implementing the QbTest, CAMHS sites scheduled more clinical appointments than Paediatrics, but there was an equal number of days from referral to diagnosis between the two types of service (see Table 1). After implementing the QbTest, CAMHS sites experienced a small decrease in number of appointments and a considerable increase in days from referral to diagnosis, while Paediatrics experienced a decrease in both areas. Both sites reported an increase in days from first appointment to diagnosis after implementing QbTest, but this was larger for CAMHS sites (see Table 1).

#### High- and low- rate testing sites

The average number of QbTests conducted per month at each site varied from around five tests to over 100. As such, descriptive comparisons were made between sites using QbTest more often (*n* = 5 sites, ≥ 20 tests/month) and less often (*n* = 15 sites < 20/month) sites. There were CAMHS and Paediatric sites in both categories. Pre-QbTest implementation, compared to low-rate sites, high-rate testing sites had a higher mean number of appointments to diagnostic decision, a lower mean number of days from initial referral to diagnosis, and a slightly lower number of days to reach a diagnostic decision.

Post QbTest implementation, higher-rate testing sites saw a considerable decrease in number of appointments to diagnostic decision, whereas lower-rate testing sites saw little difference. Similarly, higher-rate testing sites saw a decrease in number of days from referral to diagnosis, whereas lower-rate testing sites reported an increase on this metric. In contrast, higher-rate testing sites saw an increase in number of days from first appointment to diagnosis, whereas lower-rate testing sites reported a small decrease.

#### School assessments

Across sites, there was a 17% reduction in the number of school observations that were conducted post QbTest implementation (pre QbTest: *n* = 120, 26% of cases used a school observation; post QbTest: *n* = 49, 9% of cases used a school observation). CAMHS sites had more of a reduction in school observations (pre QbTest: *n* = 112, 34% used a school observation; post QbTest *n* = 39, 12% used a school observation; 22% decrease) compared to Paediatric services sites (pre QbTest: *n* = 8, 4% used a school observation; post QbTest *n* = 10, 5% used a school observation; 1% increase).

### Survey

#### Patient/Carer

Healthcare staff were responsible for distributing the survey to patients/carers, as such it is not known how many patients/carers were asked to complete it. Due to data protection regulations, it was not possible to identify the healthcare Trust the patient was receiving care from and so we do not have information about the geographical spread of survey responses. Of the 30 participants who started the survey, eight responses remained incomplete, and therefore data from 22 respondents was analysed.

As evidenced in Table 2, most patients/carers found the QbTest helpful, not difficult to complete, and reported that it helped them to understand their symptoms and how the clinician reached their decision.

**Table 2:** Patient/carers’ (*n* = 20) opinion on the clinical utility of the QbTest; % data (n)

Survey Item	Strongly Agree-Agree	Neither Agree/Disagree	Strongly Disagree-Disagree
The QbTest results helped me to understand my symptoms	50% (11)	32% (7)	18% (4)
The QbTest results were difficult to understand	9% (2)	50% (11)	41% (9)
Overall, the experience of the QbTest was helpful	68% (15)	18% (4)	14% (3)
When the clinician talked through the results with me, it helped me understand how they reached this diagnosis	41% (10)	32% (7)	27% (5)
I found the test difficult to complete	14% (3)	41% (9)	45% (10)

Analysis of the two open-ended questions revealed key themes surrounding positive elements of the QbTest including improved understanding of symptoms and diagnosis, and improved experience of the assessment process.

It helped me to see where my child’s difficulties lay as I was initially hesitant that my child had ADHD and thought her presentation was explained by other reasons, so it helped my understanding as to why school and Dr thought ADHD may be present.

Key themes surrounding negative elements of the QbTest included a lack of understanding of the results, difficulties for the child in sitting still during the test, and technical issues with the QbTest equipment.

I was not given any information about the results, this is a repeated visit. I don’t know or understand the scoring.

Some respondents also commented that they think the QbTest should be standard practice for all ADHD assessments in England.

#### Healthcare professionals

It is not known how many healthcare professionals were sent the survey, as it was distributed by site managers. 65 respondents gave consent and completed the survey. Six of the questions were not completed by all 65 respondents (see Table 3).

**Table 3:** Healthcare professionals’ opinions on the clinical utility of the QbTest; % data (n)

Survey Item	Strongly Agree-Agree	Neither Agree/Disagree	Strongly Disagree-Disagree
I found the QbTest results helpful to my understanding of the client’s symptoms (*n* = 65)	92% (60)	5% (3)	3% (2)
The output improved my communication with clients (*n* = 65)	71% (46)	22% (14)	7% (5)
I found the output difficult to understand (*n* = 65)	15% (10)	28% (18)	57% (37)
The test is not a good use of clients’ time (*n* = 65)	5% (3)	12% (8)	83% (54)
QbTest was easily incorporated into the assessment protocol in our clinic (*n* = 65)	86% (56)	7% (5)	7% (4)
QbTest results would be best shared prior to the clients contact with clinician to reduce the length of an initial consultation interview (*n* = 65)	40% (26)	22% (14)	38% (25)
QbTest should be routinely used as part of the diagnostic assessment (*n* = 65)	58% (38)	27% (17)	15% (10)
QbTest should be reserved for use in cases where there is diagnostic uncertainty (*n* = 64)	39% (25)	17% (11)	44% (28)
QbTest makes it easier to explain to a patient why they do not have ADHD (*n* = 64)	73% (47)	19% (12)	8% (5)
QbTest makes it easier to explain to a patient why they have ADHD (*n* = 64)	84% (54)	14% (9)	2% (2)
QbTest results were supportive when evaluating treatment effects (*n* = 62)	39% (24)	60% (37)	1% (1)
QbTest results were supportive in the discussion with the patients at the follow-up visit (*n* = 62)	60% (37)	37% (23)	3% (2)
The results influenced my decisions about treatment (*n* = 63)	33% (21)	51% (32)	16% (10)

The survey results in Table 3 demonstrate that respondents rated QbTest positively. Most agreed that the QbTest results were helpful in understanding their client’s symptoms and improved communication with the patient, and that the test was easy to implement in their practice. Most respondents said they neither agreed nor disagreed with the statement that QbTest results support evaluation of treatment effects. These responses were clarified in a free text response, where respondents explained that they were not using the QbTest for this purpose. However, when used for this purpose, respondents reported greater confidence in decision making from using an objective measure to evaluate medication outcomes.

There was more heterogeneity in opinion as to whether QbTest should be used in all cases or reserved for complex cases with 39% strongly agreeing or agreeing that it should be used only when there is diagnostic uncertainty, and 44% strongly disagreeing or disagreeing with this. Furthermore, opinions were more divided on whether the QbTest results should be shared with patients before their contact with a clinician with 40% strongly agreeing or agreeing that QbTest results should be shared in advance and 38% strongly disagreeing or disagreeing with this. Responses to the open-ended item revealed some cases where QbTest was less useful, namely complex cases where patients had significant functional impairments. Specific characteristics were identified which were thought to make cases more suitable for QbTest assessment, including female patients and older children. Additional advantages included: identifying patients who should be referred for autism assessment more quickly once ADHD diagnosis was ruled out, identifying patients who mask ADHD symptoms at school/home, an additional source of information in cases where there is diagnostic uncertainty after using other assessment methods, and in cases where the parent or school does not agree with the diagnostic decision and/or whether treatment has been effective.

### Interviews

Data saturation was reached after the 20th interview, with data collection stopping after 22 interviews. One interview was not properly recorded and therefore could not be transcribed, as such 21 transcripts were included in the analysis. Themes, subthemes, and illustrative quotes are presented in Table 4.

**Table 4:** Themes, subthemes and example quotes from the interviews with healthcare professionals

Framework Category	Themes	Subthemes	Example Quotes
**Patient Experience**	***Provides a Faster and More Efficient Diagnostic Process***	Increased understanding of the self and symptom profile	*It’s really nice for parents and for the young person to understand about themselves and have that objective measure to look at to show that* *I think for young people*,* as well*,* they feel really a part of their care*,* because they’re actively doing something*,* and then they’re able to see the report and talk about it*,* rather than having information about them gathered by other people*,* which is how it used to be. There wasn’t much asked from the young person. The young person was observed*,* but information would be gathered from schools*,* and parents*,* and observations*,* whereas now they can be really involved.*
		Individuals with subtle presentation more likely to be accurately diagnosed	*Sometimes they just go under the radar at school*,* don’t they*,* particularly the girls with attention problems that are just quiet and getting on with it at the back of the class*,* where school are saying there’s no problem*,* and then they come*,* and they have a QB that shows they’re really struggling*
		Shorter pathway from referral to diagnosis	*I see it as a way of reducing the amount of time children are waiting to be seen. And thus*,* reducing the number of follow-ups*,* thus reducing the number of times they have to come back to the hospital so it’s an opportunity to save the patients and parents’ time.* *I think for a lot of families*,* it’s the anxiety of knowing or not knowing. And the length of time they have to wait to get a diagnosis*,* the fact that Qb can reduce that length of time*,* that’s the important factor to me*
		Faster diagnosis led to faster implementation of educational support	*I think it’s helpful to have concrete information to say to schools*,* actually you need to put in support*
	***Objectivity of Tool Supports Patient Understanding***	Tools objectivity supported clinician justification	*I think parents themselves like the fact it’s not based on one person’s perceptions*,* its actually based on something that is measurable and understandable*
		Supported medication titration, and assessment and communication regarding medication utility	*We get a lot of people of adolescent age that say ‘oh I don’t want my meds’ and have come off the meds and then deteriorated*,* and actually if you can do those baseline results with them*,* I think it will just give us something evidence-base to start having those conversations of ‘actually do you need medication/do you not need medication/what are the benefits that medication bring*
	***Patient Difficulties and Exclusions***	Sensory discomfort during QbTest	*A lot of our young people that come in for both an autism and an ADHD assessment can experience difficulty with the plastic covering of the headband*,* because it’s quite a sensory thing on the head and that can be quite uncomfortable. It’s quite tight on the forehead and around the head*
		General and anxiety during QbTest	*Some young people were not able to complete the Qb itself. […] After a couple of practices*,* they feel too frustrated to continue or they feel too anxious to use the responder button after the video instruction has been presented*
		Difficulties with younger children and older adolescents during QbTest	*The only thing where it might not suit a patient are perhaps the very*,* very young ones*,* so maybe the 6 or 7 year olds. It’s doable*,* because we have done them*,* but we often find we have to rephrase a lot of the instruction video*,* I think it sometimes confuses them a little bit*,* but we rephrase and practice and it’s fine*
		Exclusions from the use of QbTest	*[If a young person has] a disability or very*,* very high anxiety*,* they’re the only times we might then do something different instead*
**Clinician and Service Experience**	***Benefits of an Objective***,*** Evidenced Based Tool***	Provided confidence to staff during diagnosis, especially for cases with subtle presentation	*I think it gives all clinicians a bit more confidence around making diagnosis*,* and I think for nurses*,* that’s where its particularly helpful. Especially if they’re nurse prescribers*,* because they have that responsibility of making the diagnosis and providing medication. So*,* they want it to be… they want to feel very*,* very sure that this is ADHD*,* that nothing is being missed*
		Provided useful additional information for assessing complex cases	*I think it works well with subtle presentations. Presentations maybe where there’s a disagreement between school and home. Cases where there are parental disagreements. Cases where young people themselves are unsure* *The really complex ones*,* it takes months. But QbTest is showing that doesn’t have to be the case now*
		Supported clinicians in communication with families	*I think they offer a very visual result for the parents*,* […] especially the little chart that shows hyperactivity and stillness and the wild swinging round. So*,* I think that sort of aspect to it is really good to be able to communicate the diagnosis* *Sometimes parents find it difficult if we do say that it’s not a diagnosis for ADHD*,* because then you often will get the ‘oh*,* well they like computers and they like computer games*,* so they must have liked the test*,* and that’s why the results are what they are*
		Useful for medication reviews	*I find that helpful*,* particularly when parents may be saying ‘oh his medication isn’t working’. I’ll often do a QbTest then both on and off medication to get results*,* I find that helpful*
	***Supports a Faster and More Efficient Diagnostic Process***	Supported a diagnostic decision being made faster and with fewer appointments	*The Qb is supporting the diagnostic decision and actually strengthening the decision-making process*,* so that that psychiatrist can make a decision there and then as to whether it’s yes or no*
		Supported formulation; reduced need for reformulation	*It’s really reducing the number of children that are going back to be reformulated. […] Now what we’re finding is that because the QbTest is there*,* is that a decision is made on that day rather than having to take the child back for formulation and re-discussion*
	***Time and Cost Saving***	Fewer school observations, shorter appointments	*Actually*,* we don’t always ensure the children have a school observation if we’ve got all of the Conner’s and we can do a Qb. Sometimes we don’t necessarily require school observations*,* so actually it saves some appointment times as well* *In the time that we do the school observation*,* we can do two or three Qbs*
		Delivered by other staff in a multi-disciplinary team	*In terms of administration*,* it’s suited to someone like me on a junior level rather than a consultant*
	***Difficulties identified when delivering QbTest***	Language use within QbTest	*They’re looking around the room and asking when it’s going to finish*,* ‘this is boring*,* need to finish this now*,* it’s not a game*
		Issues identified with use of QbTest in diverse populations	*I’ve had some discussions with the people from Qb because we’ve had a few transgender young people come into our assessment*,* pathway for an assessment and when you’re trying to set up a profile for that young person you have to identify the biological sex*
**Implementation factors**	***Reasons for Adoption of QbTest***	Reduced waitlists for ADHD assessment	*I wanted to improve the patient experience. I wanted to shorten the pathway for them*
		Positive feedback from other Trusts & clinicians	*I felt it would be of benefit to our Trust because it has such as impact on other services where it was in use*
	***Implementation Support***	Importance of Trust support	*We have regular monthly team meetings where*,* like how we do this was discussed with everybody in the team and so there was collective decision making in terms of its implementation*
		Importance of Qbtech Ltd. Support	*The support that you get from Qb if there are difficulties with interpretation is just really accessible for us to use during those incidences*
	***Technological and Logistical Challenges***	Room requirements for QbTest delivery	*The main [challenges] were just the practical side*,* like the room space and things. It’s really competitive to get rooms here so making sure it was booked well in advance*
		Equipment and Wi-Fi required for QbTest delivery	*We don’t actually have our own Qb kit as a team. […] The main challenge was accessing the laptops and making sure the password was shared between the sites*
	***Factors Affecting Continued Use***	Adaptations to QbTest delivery	*My long-term plan was obviously always to get it to the front of the pathway where it’s meant to be. Which is where we have now recently put it*,* and changed our pathway*
		Staff capacity	*The only problem we have is there is more demand than there is capacity*
		Funding	The extra funding from AHSN has been *the* factor in allowing us at the moment to get it into the front pathway. And I hope that this time period will then enable us to demonstrate to commissioners that actually it is something that we need in the long term

#### Framework 1: Patient Experience

Analysis of the question ‘what is the patient experience of QbTest?’ revealed three key themes and 10 subthemes. Two themes focussed on the positive elements of the patient experience, including providing a faster and more efficient diagnostic pathway associated with enhanced understanding of symptoms, more accurate diagnosis of subtle symptoms, and shorter pathways from referral to diagnosis leading to faster implementation of educational support. The second positive theme related to the objectivity of the assessment process that supported families’ understanding of the ADHD process and acceptance of the diagnostic decision. Specifically, caregivers welcomed the corroboration of the QbTest report alongside clinical judgement and the QbTest results supported acceptance around the decision-making process involved in medication. The third theme highlighted caregivers’ negative views of the QbTest, including patient sensory discomfort (i.e., from the headband) and anxiety/difficulties around following/understanding the task instructions, particularly for younger children (e.g., the 6–7-year-olds).

#### Framework 2: Clinician Experience

Analysis of the question ‘what is the clinician and service experience of using the QbTest?’ revealed four key themes and a total of 10 subthemes. Three of the four themes related to positive experiences of using QbTest. These included, the benefits of an objective, evidence-based tool supporting increased clinical confidence during diagnosis, particularly for cases with subtle or complex presentations, and for supporting communication with families, especially during medication reviews. The second positive theme related to the efficiency of the diagnostic process reducing number of appointments and the need for reformulation. This leads to the third positive theme, surrounding the reduction in school observation and appointment length, as well as the fact the QbTest could be delivered by other staff (e.g., assistants) within a multi-disciplinary team. The negative theme related to issues surrounding patient expectation of conducting a QbTest, and clinicians needed to be mindful of the language they used. Young people struggled with the use of the term “test” in the explanation video, becoming stressed at the prospect of being tested. While avoiding the word “test” helped to alleviate stress for the young people, it also appeared to be important to also avoid the word “game”. This helped to avoid a mismatch in expectations between what the young person thought was going to take place, and the content of the QbTest. In addition, there were concerns about using QbTest in diverse populations. One site noted that as most of their young people coming for assessment were Bangladeshi, the explanation video did not represent them or people they might typically interact with. Another site also noted that during the creation of the young person’s profile they had to choose biological sex, which may not be suitable for transgender young people.

#### Framework 3: Implementation

Analysis of the question ‘what factors are important as barriers and facilitators in the implementation of the QbTest into clinical settings?’ revealed four key themes and a total of nine subthemes. The first theme related to the rationale for adoption, and this reflected the NASSS domain of ‘value proposition’ relating to the expected benefits of the QbTest compared to the costs. Key motives for adopting the QbTest appeared to be reduced waiting lists and positive feedback from other sites.

The second theme related to the need for support to implement QbTest. Sites discussed the support they had received during the implementation of the QbTest which provided enhanced capacity to innovate and readiness for change, reflecting the NASSS domain of ‘Organisation’ in supporting adoption. Most sites found the support from Qbtech Ltd. during the implementation, set-up, and during on-going use to be accessible and helpful. They found the initial training on how to set up and use the QbTest to be useful and in-depth, and there were also refresher training sessions offered. Qbtech Ltd. were also available for on-going support with interpreting complex output reports. This was useful for complex cases, and for checking the reliability of the test with different co-occurring conditions. However, not all sites seemed to be aware of the on-going support available to them. Staff also found that communication from their managers during the implementation process was useful so that everyone knew what was happening and why.

The third theme related to technological and logistical challenges. This also reflects the NASSS domain of ‘Organisation’, as these were not related to the QbTest technology itself, but rather the additional requirements needed within the organisation, including having a permanent room to prevent disassembling and reassembling QbTest equipment, issues with sharing the required kit and passwords, as well having sufficient Wi-Fi access.

The final theme related to the feasibility of continuing to use and adapt the technology within the service, reflecting factors related to NASSS domain on ‘embedding and adaptation over time’. Since implementing the QbTest, sites either had made, or planned to make, adaptations to the way the QbTest was administered within the pathway. These adaptations included positioning the test earlier in the pathway so that the results were available at the first appointment with an ADHD clinician. Some sites made, or wanted to make, changes to who delivered the QbTest, namely enabling junior members of the team to deliver the test to release nurse time. Staff capacity was another factor influencing the on-going use of the QbTest. The demand for QbTests exceeded some sites’ capacity to administer the tests due to a lack of staff. Furthermore, some sites found that the use of the QbTest caused a larger waitlist for medication prescription as, while they had enough staff capacity to deliver the QbTest, this was not matched by the capacity for medication prescription. Funding was another factor determining the continued use of QbTest. Some sites had used the QbTest previously but were unable to continue once the funding ended. Other sites credited extra funding as the reason they were able to continue using the QbTest. Funding also affected staff capacity, as without sufficient funding to hire more healthcare staff, the site could not conduct the number of QbTests requested.

## Discussion

With the aim of providing an independent evaluation of the Focus ADHD national programme, this report outlines the impact of QbTest on the time taken to reach a diagnosis, exploring feasibility and acceptability to families (patients) and clinicians within CAMHS and Paediatric NHS sites in England. The findings represent the first national evaluation of QbTest and demonstrated small service efficiencies in terms of a small reduction in the number of appointments to diagnosis and positive feedback from healthcare staff, however there was an increase in number of days to diagnosis and benefits were greater for paediatric services.

The primary evaluation compared number of clinical appointments to reach a diagnostic decision on ADHD pre and post QbTest implementation and demonstrated a reduction in the average number of clinical appointments by 11.5% or 0.37 appointments. Across the whole dataset, There was a large increase in the average number of days from initial referral for ADHD assessment to reach a diagnostic decision (55 days), although further review found this increase was predominantly driven by CAMHS sites. We found a smaller increase (12 days) in the number of days from first appointment with a clinician to reaching a diagnostic decision. Separate exploration of case-note data from CAMHS and Paediatric services revealed that Paediatric services benefited from a greater reduction in clinical appointments compared to CAMHS. While school observations were reduced by 22% in CAMHS, there was little difference in Paediatrics services. Furthermore, sites that had a higher rate of using QbTest showed a 26.8% decrease in number of clinic appointments, whereas lower-rate sites stayed approximately the same. The qualitative findings from surveys and interviews indicated that patients and clinicians are generally positive about the use of QbTest, but identified some areas of concern. Here, we interpret the findings of the quantitative data in light of the context provided by the qualitative data.

The reduction in appointments was less than previously found in research and studies [15, 22]. It Is possible that the difference reflects a flattening effect when QbTest is evaluated on a larger scale, this effect has been found in other studies and may result from a loss of quality in implementation [23]. However, given AQUA-Trial was also a multi-site evaluation, we are uncertain if this was the case. One alternative explanation may be that in this evaluation, post-implementation case-note data was the first 10–30 patients to have completed a QbTest. Thus, the data is from a period where each site was a new user of the QbTest and unlikely to be fully proficient at integrating it into their clinical decision making. Our findings also reflect the impact of COVID-19 on services. This evaluation was conducted during 2021–2022, with sites typically drawing pre-QbTest data from pre-COVID-19 and post-QbTest in the aftermath of COVID-19. During COVID-19, shorter and more frequent telephone or videoconference appointments were conducted. As telephone appointments alone cannot provide objective clinical assessment, it’s probable that in some select cases (e.g. areas of uncertainty, complex cases) additional face-to-face appointments (when allowed) might have been required to complete the diagnostic process. A combination of these factors is likely to have increased the number of clinical appointments required for a diagnostic decision to be made in the COVID-19 period. Despite noted difficulties in service provision and efficiencies during this demanding time, it is perhaps equally as impressive that any reduction was noted.

The increase in number of days to diagnosis after QbTest is broadly in line with the finding from the AQUA-Trial [15], which found number of days to diagnosis was not a sensitive measure of QbTest impact. Given that days to decision is influenced by availability and attendance of appointments, it could be argued that this is not a useful measure of QbTest clinical utility. Although we believe that waiting times (in terms of days) are an important metric, it is likely that waiting times are influenced by a combination of multiple wider healthcare system variables that QbTest is not designed to support (including number of appointments available, funding strategies, release of appointment times and non-attendance). Our qualitative data showed that during COVID-19 lockdown, ADHD appointments were de-prioritised and clinical assessments were remote. QbTest must be completed in-person, there is therefore an inbuilt risk of increased delays due to QbTest scheduling requirements. Alternatively, although we consider this unlikely, it cannot be ruled out that QbTest is a factor in the increase of days and this would be something to investigate further in future evaluations.

Of particular note, Paediatric services had almost double the reduction in number of clinical appointments to diagnosis compared to CAMHS, despite a comparatively lower number of clinical appointments at baseline (pre-QbTest). Our conversations with key NHS staff and decision makers felt this finding may reflect a differential impact of COVID-19 between CAMHS and Paediatric services. Specifically, CAMHS may be more likely to see a greater variety of complex and co-morbid presentations, which may have increased during COVID-19 [24] and discussion with our clinical advisors indicated that staff from CAMHS were more likely to be re-assigned to front-line COVID-19 services. Further exploration indicated the reduction in clinical appointments for Paediatrics was unlikely to be explained by reducing school observations. School observations were reduced by 22% in CAMHS, while Paediatrics stayed stable. However, it is also likely that the metrics used in this evaluation did not fully account for the time and costs savings that would result from reducing school observations. As noted in our qualitative interviews, school observations take approximately 4 h and are associated with additional travel costs. This evaluation did not account for length of appointments and did not conduct a budget impact assessment; thus, it is possible that CAMHS benefited by reducing appointment minutes rather than number of appointments.

Comparing usage metrics showed that sites which were higher-rate users of QbTest showed a 26.8% decrease in number of clinic appointments, while there was no change for lower-rate users. This may reflect that higher-rate users had more experience with QbTest, resulting in the system being better embedded in the service pathway and clinicians being more proficient in interpreting the test results. However, we also observed an increase in days from first appointment to diagnosis in the frequent testers, which is not seen in the less frequent testers. We have discussed above how “days” may not be the most sensitive metric to assess the QbTest, reasons for this should be explored in future evaluations.

When interpreting the impact of the results reported here, it should be noted that diagnostic accuracy was not evaluated, thus we cannot state whether reductions in time to diagnosis reduced the accuracy of the decision making. Data from the AQUA-Trial [15] showed that adding QbTest did not reduce diagnostic accuracy, despite significant decreases in time to decision. Furthermore, there is no evidence that explores whether QbTest can act as a proxy for school observations. Thus, despite resulting in likely cost and efficiency savings, it is possible that by removing these ‘real-world’ observations, important diagnostic information is missed.

**Fig. 2:**
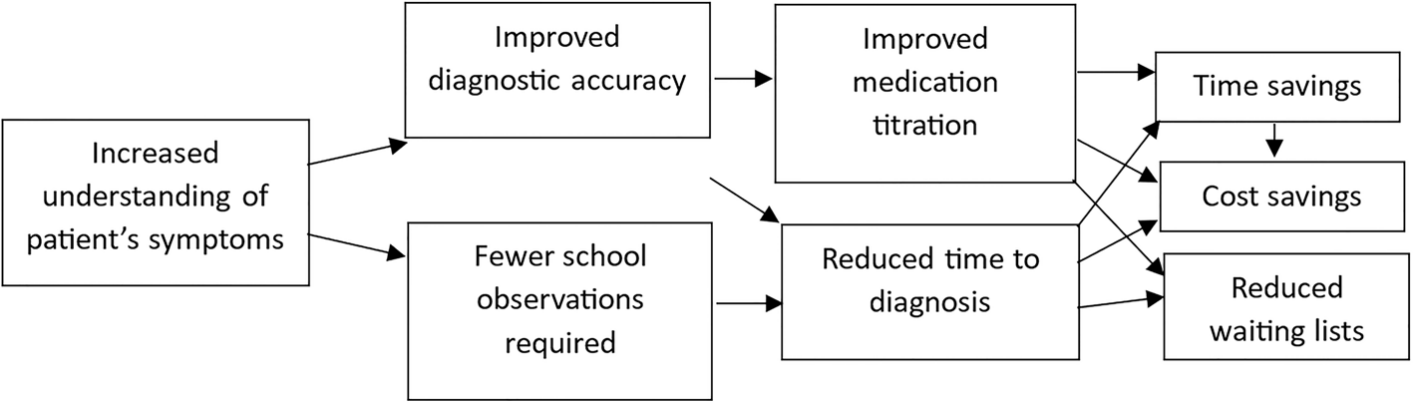
Pathway to improve efficiency in the ADHD assessment process

The qualitative interviews revealed rich data exploring possible mechanisms by which QbTest facilitated a more streamlined and efficient process. Evaluating these mechanisms quantitatively was out the scope of the evaluation. However, future research could explore the impact of QbTest on the whole pathway. We highlight in Fig. 2a suggested pathway of impact based on the qualitative data reported here.

A number of implementation factors were identified as part of this evaluation. These are summarised in Table 5.

**Table 5:** QbTest implementation factors

Implementation Factors to Consider
Age of the patient (more challenging for < 7years)
Managing patient anxiety relating to being ‘tested’
Sensory sensitivity of the patient (due to wearing the headband)
Utilise Qbtech support for technical and clinical advice
Quiet, reserved testing room required
Scheduling of shared use of the equipment (where applicable)
Good Wi-Fi connection
Staff capacity for testing – consider training more junior staff

The learning from this evaluation should be utilised to improve future QbTest implementation and facilitate more effective QbTest practice in clinics which already make use of this assessment tool. Specifically, it would be interesting to conduct a longer evaluation to explore whether seasonal changes (including school holidays) or Trust specific factors (e.g. size of region, number of patients) determined whether sites were high or low users of QbTest and how this impacted the benefits of using the Test.

There were significant barriers and limitations to this evaluation, including the influence of COVID-19 on standard practice, and Trust resources for both participation in the study and delivery of ADHD assessments. As such, a follow-up evaluation following the COVID-19 health pandemic should be conducted. A further limitation is that sites were required to use data from their first QbTest cases to reduce the time window between pre- and post-test implementation and minimise the confounding influence of other general changes in practice/state of the health system. However, it is possible that data from established QbTest users would provide different conclusions. Given that the greater potential for benefit of an intervention is likely to be realised once it is embedded into a service, it is possible that our findings underestimate the potential benefit of QbTest. There is also a possibility of a non-response bias from sites. For example, sites which participated may have been those which had greater resources and were less affected by COVID-19 or were more motivated to demonstrate the benefits of the QbTest. Indeed, the top two reasons for non-participation were “no capacity to participate”, due to COVID-19 or staffing issues, and “unable to provide enough cases” (either pre or post QbTest implementation). Although all participating sites met the minimum requirement of 10 pre and post cases, seven sites were unable to make the target of providing 30 pre and post cases. Finally, sites were responsible for their own data collection and the evaluation team were not able to check for accuracy against the raw data (e.g. clinical file), and as such, errors in reporting may have occurred. To mitigate any potential errors or biases, sites were trained by the evaluation team and all data was checked for missing data or inconsistencies, and these were rectified by communication. These limitations may influence the generalisability of the findings, and we would recommend further real-world evaluation as part of on-going service improvement.

Although, neither treatment initiation nor the impact on waiting lists was explored in this study, the role of QbTest in the whole service pathway should be explored in further research to understand if reducing the number of appointments to make a diagnosis results in whole service efficiencies. To maximise the use of QbTest as part of routine clinical practice a key next step is engage service managers, providers, and commissioners to discuss the barriers identified here surrounding resource capacity (in terms of clinical time and equipment required) to support appropriate resource planning to aid implementation.

## Conclusion

The evaluation of the Focus ADHD national programme demonstrated that implementing QbTest as an adjunct to standard clinical practice on a national scale resulted in a small reduction in the number of clinical appointments required to reach a diagnostic decision of ADHD, with greatest benefit demonstrated in Paediatric sites. However, the evaluation was impacted by COVID-19, which is likely to have negatively skewed possible benefits. The QbTest was received positively by healthcare staff, patients, and their families. The findings indicate the clinical utility of embedding QbTest in the ADHD care pathway.

## Supplementary Information

Supplementary Material 1.

## Data Availability

The datasets generated during the current study are available from the corresponding author upon request.

## Abbreviations

ADHD: Attention/deficit hyperactivity disorder
HIEM: Health Innovation East Midlands
AHSN: Academic Health Science Network
CAMHS: Child and Adolescent Mental Health Service
NASSS: non-adoption, abandonment, scale-up, spread, sustainability
NHS: National Health Service
NICE: National Institute for Health and Care Excellence
