# Supplementary material for: A national evaluation of QbTest to support ADHD assessment: a real-world, mixed methods approach
Source: BMC Health Serv Res. 2024 Oct 8;24:1201. doi: 10.1186/s12913-024-11693-7 (PMC11459991; doi:10.1186/s12913-024-11693-7)
Supplement: Supplementary file 1 — Supplementary Material 1. [file 12913_2024_11693_MOESM1_ESM.docx]

**Appendix A - Interview Schedule**

**Focus ADHD national programme evaluation V1.2 21.05.21**

Researcher goes over Focus ADHD national programme evaluation, reminding the clinician what the purpose of the project was.

To assess how well this actually works in real practice it is important that we listen to what clinicians have to say about this – and that includes you! It is important that we get everyone’s perspective on this project and you are crucial part of this. We genuinely want to know how you feel about this project and we can use your information to help us develop further projects.

I’m recording it so I can fully concentrate on what you say and not have to write notes as we go along. I want you to pretend that I know nothing about this project when I ask you the questions. I want you to answer honestly.

**General Background Questions**

1. Can you give me a little information on your professional background

- How long have you worked in CAMHS/Community Peds
- training
- Profession/role

1. How would you normally assess ADHD cases?

- Scales, measures, ask young person?
- Use QbTest
- How long would it take and how many visits

1. How many times have you used QbTest?

- Rough idea – lots, only a few.

**General QbTest Questions**

1. Did the QbTest help or interfere with you carrying out your normal duties?

These next questions are about your own use of the QbTest within your own individual practice.

1. Why did you decide to be involved in QbTest?

- Like QbTest (maybe use it anyway?)
- Manager pressure
- Wanted to help

1. How did you feel about being involved/using QbTest?

- Did you know what was expected of you?
- Were you given training in ….?
- Did you have concerns about the time it took to complete?
- Did you have concerns with the technology used to complete it?

1. How do you feel about using the QbTest now?

- Has it run better / worse than you thought?
- Have you had any problems?

1. How difficult or easy is the test to use for clinicians and patients?
2. What helped you use it?

- Members of admin support. Other clinician support. Manual. Researcher. Time. Young Person/parent support, anything else?

1. What hindered you using the QbTest?

- Negative attitude from YP / other admin. Lack of time. Equipment not working, IT and IG requirements, anything else?

1. What changes to the existing system and procedures were needed to facilitate your use of QbTest?

**More detailed QbTest questions**

1. Did it help you understand your clients symptoms?

- How? Why? Which ones?

1. Did the output help with communication with the client?

- How? Why? Any examples? Particular cases?
- Does it help explain if they DON’T have ADHD
- Does it help explain if they DO have ADHD

1. Do you think the test is a good use of time?

- Why?

1. When do you think would be the best time to do the QbTest?

- Before session? After? Another day? and how often?

1. Do you think they should be used routinely as part of diagnostic assessment?

- Why? If not routine, in which cases?
- Or only for cases of uncertainty? Or would you see it as routine use? Why?

1. Did it influence your treatment decisions?

- Sometimes? Always? Never? What cases and why?

1. Who does this test work well with, in terms of patient and clinician characteristics?
2. What value does the QbTest add to the clinicians/patient/families/the ADHD diagnosis system locally?

**Adoption of the QbTest by your Clinic**

These questions are about the wider adoption of QbTest within the whole clinic/setting.

1. Do you think the use of QbTest is running well in this clinic?

- Why or why not?

1. Who was the person/persons responsible for implementing the QB test at your site?

- What is their job role(s)?

1. Were there any challenges to implementing the QbTest at your site?

- What were they? How did you overcome them?
- E.g. Organisational culture, resistance to change; anything related to the existing setting and systems/procedures, IT systems. Where was support lacking in the system

1. What helped facilitate implementation?

-Organisational culture, anything related to the existing setting and systems/procedures, IT systems., Where was support experienced in the system?

Thinking back to when the QbTest was first adopted in the clinic

1. What were the reasons you decided to implement the QbTest early/later?
2. What information did you need to enable implementation at your site?
3. How was communication managed in regard to implementation?

- What worked well? What didn’t?

1. Did you feel supported in implementing the QbTest? Both from your site and the QbTech team?

- What was the impact of having/not having enough support?

**Future of QbTest**

1. Would you recommend it was used routinely in clinics?

-Why? Is it easy to integrate

1. Would you recommend it being used routinely in other clinics or other settings?

-Why?

1. If QbTest was to continue as part of routine clinical practice what suggestions would you make to improve the process?
2. Do you think you’ll be able to maintain using the QbTest indefinitely?

- Why or why not?
